# Vaccination in the Light of the Royal British Commission

**Published:** 1897-08

**Authors:** M. R. Leverson

**Affiliations:** Fort Hamilton, L. I., N. Y.


### VACCINATION IN THE LIGHT OF THE ROYAL BRITISH COMMISSION.

M. R. LEVERSON, M. D., FORT HAMILTON, L. I., N. Y.

(Continued from page 253.)

Brigade Surgeon Dr. Wm. Nash began his testimony on the 8th November, 1889 (page 111 of the 2d report of the R. B. C.).

He stated that every recruit since 1858 was vaccinated or revaccinated, whether he was consenting thereto or not till 1883, but from that date a question was introduced into his attestation paper, "Are you willing to be vaccinated or re-vaccinated?" (Qs. 3,450-5.) If he has been vaccinated before he is still required to be revaccinated (Q. 3,457).

The "lymph" is obtained by preference from arm to arm, but not from adults nor from a revaccination (Q. 3,461). Not less than two punctures are insisted on in cases of revaccination, but "where the evidence of original vaccination is indistinct or single, three punctures will be made" (3,462).

In (Qs. 3,463-6) are explained the various measures taken to prevent the escape of a single victim. At (Q. 3,467-8) Dr. Nash hands in two tables (App. No. 8, Tables A and B, page 278, R. B. C.) showing the number of primary vaccinations and revaccinations of soldiers and recruits, and of women and children pertaining to the army and under the care of the army medical officers.

At (Qs. 3,467-3,475) Tables C and D are handed in showing the average annual strength of the army during the twenty-nine years, 1860-1888; the number of admissions into the hospitals of small-pox patients among the white troops, and the deaths in each year, together with the ratios per 1,000 in each year; also admissions and deaths from small-pox in the army in the United Kingdom, in the colonies, India, and Egypt respectively, and the ratios per 1,000 of strength.

These tables are instructive, and I should like to reproduce them, but as very few would care to wade through them an abstract, setting forth their substantial results, will probably be more interesting to the readers of THE HOMŒOPATHIC PHYSICIAN, though very troublesome to calculate and extract for their benefit.

From Table C we learn that during the above twenty-nine years there were 3,953 cases of small-pox among the white troops of the British army, and that 391 of them died. The yearly average strength of those troops, calculated from Table C, was 177,868, the yearly average of small-pox cases was 140 and 13.5 deaths. Thus the average of cases is .078 per cent. of strength, and the deaths .07 per 1,000 living.

But small-pox is an epidemic disease, and in 1860 the number of cases was 329, with 30 deaths, or .16 cases per cent. of strength with .15 small-pox deaths per 1,000 living. In 1861 there were 331 with 43 deaths, or .17 cases per cent. of strength, with .22 per 1,000 deaths. In 1864 there were 325 cases with 30 deaths, or .17 per cent. of cases and deaths .19 per 1,000 living. In 1871 there were 329 cases with 36 deaths, or .19 per cent. of cases with deaths .21 per 1,000 living. From Table D we learn that in India during those twenty-nine years there were 1,840 cases with 227 deaths. In the year 1861 there were 226 cases with 35 deaths, or 4,000 cases per million, and 610 deaths per million living, while in Egypt in 1884 there were 25 cases with one death. In 1885 there were 52 cases with four deaths, or 5,400 per million cases, with 420 deaths per million living, while in 1887 the cases were 4,900 per million, and 760 deaths per million living. In 1888 there were 4,200 cases per million, with 1,190 deaths per million living.

Now the whole army had been thoroughly vaccinated and revaccinated, in many cases re-re and re-re-revaccinated since the order of 1859 went into operation. Dr. Nash admits (Q. 3,542) that the army was well vaccinated, and, as he hopes, thoroughly so; also (Q. 3,543) that it is as well vaccinated as any other part of the population, yet the incidence of small-pox in the army was very much greater than among the civil population of Great Britain, Ireland, or Scotland, greater even than among the civil population of London.

It should be remembered, too, that the army consists of men picked for health and vitality, and in the most virile time of life.

Dr. Nash, in explanation, points out (Q. 3,544) that of 39 cases in 1879, 33 (according to Table C, 32) occurred in India, and being thereupon asked by Mr. Picton (Q. 3,545), "Then does vaccination fail to protect in India?" replied, "No, I do not think so, but in India there is always a great amount of small-pox among the people." (Q. 3,546.) "But are we not right in supposing that vaccination is intended to protect against contagion?" "Yes, certainly." (Q. 3,547.) "Then it did not protect in India in this case? It did not confer perfect immunity evidently."

(Q. 3,549-52.) It was not because of any imperfect vaccination of the soldiers in India, nor was there any difference between their vaccination and that of other troops. They had all been vaccinated at home before they went to India. The explanation Dr. Nash gives for the great amount of small-pox in India is that the men are more exposed to contagion there, and (Q. 3,553) the same explanation applies to the troops in Egypt.

There is no year since 1860 in which there have been no deaths from small-pox in the British army (Q. 3,557), and in answer to the question (Q. 3,558), "Are you aware that many large populations are free from small-pox deaths for several years in succession?" he replies, "It may be so; I do not know; I have never studied the subject." And this will be found to be the fact with every advocate of the rite.

At (Q. 3,654) Dr. Nash promises to get the statistics of the small-pox attack and death-rate in the army before vaccination was introduced, and he afterward presented Table F (App. viii, page 279, of second report, R. B. C.) which is a "Table showing the admissions from small-pox among the troops in the United Kingdom during the thirteen years 1847-59, together with the ratio per 1,000 of the strength."

From this table the yearly average strength of the British army is found to have been 52,786. The total of attacks was 1,555, whereof 113 died, being an annual average of 119.6 attacks, with 8.7 deaths; and this period included the entire period of the war with Russia, called the Crimean War, and the war of the Indian rebellion.

It should be remembered that every law imposing vaccination in every community where it has prevailed has been predicated on the assertion that, "What renders the cow-pox virus so extremely singular is that the person who has been thus affected is *forever after* secure from the infection of the small-pox; neither the exposure to the variolous effluvia nor the insertion of the matter into the skin producing the distemper," and "it clearly appears that this disease (cow-pox) leaves the constitution in a state of perfect security from the infection of the small-pox." (Jenner's inquiry into the cause and effects of the variolæ vaccinæ.) As failure after failure proved the falsehood of this assertion, various excuses were introduced, first by Jenner himself, which led Cobbett to exclaim, "Quackery has always one shuffle left," and then following in his footsteps, by his *worthy* successors. But this experience of the British army falsifies them all!

At (Q. 3,516) Dr. Nash puts in the amended orders for vaccination and revaccination issued from the Army Medical Department 20th November, 1865, wherein is a description of the course of the disease, substantially the same with that given by Dr. Seaton (*Handbook of Vaccination*, pages 81-2).

But this description exactly describes the ulcers produced by *inoculation* from a syphilitic chancre. (See Ricord's illustrations of venereal disease, as arranged by Paul B. Goddard, M. D., plates II and III.)

Although "arm-to-arm" lymph has been constantly insisted on by the Local Government Board for the vaccination of the civil population of Great Britain since March, 1885, "the veterinary department at Aldershot has supplied calf lymph sufficient for all demands for carrying out vaccination and revaccination in the army" (Q. 3,466). The witness knows nothing as to the source of that lymph (Q. 3,561), but has been informed that the reports with regard to the results of using that lymph were not very satisfactory, owing, it was said, to the lymph supply not being properly arranged and being kept too long before being used. This has since been improved with satisfactory results (Qs. 3,564-6). According to the Army Medical Report for 1887 the failures in revaccination of soldiers were 280.7 per 1,000, which Dr. Nash attributes to the influence of previous vaccination, but from the same report the failures in *primary* vaccinations of soldiers and recruits were 279.4 per 1,000, and although he states that the so-called primary vaccinations may include men who had been vaccinated but had no marks he can give no further explanation (Qs. 3,570-83). This statement with regard to "men who had been vaccinated but had no marks" should be remembered when studying statistics of attacks and deaths among the vaccinated and alleged unvaccinated respectively.

Dr. Collins then turned to the Army Medical Report of 1886, where the failure of primary vaccination was 250 per 1,000, and that of revaccinations 263 per 1,000, but Dr. Nash can give no better explanation (Q. 3,584).

In the case of children the revaccinations were successful in from 750 to 850 per 1,000 (Q. 3,593), so that it seems to be more successful in the younger class (Q. 3,598). Hence it appears that what is called successful revaccination really depends more upon the age of the patient than upon the length of time which has elapsed since the primary vaccination.

In 1885 there were fifty cases of small-pox with four deaths among the British troops in Cairo (Q. 3,603), and there were a good many in 1889, although revaccination was largely practiced (Qs. 3,604-7).

In 1872, according to the Army Medical Report, there were forty-five cases of admissions to the hospitals for sickness consequent upon vaccination, and similar entries appear in subsequent years. Dr. Nash cannot explain this, but supposes that it was done to keep the soldier quiet when his arm was so inflamed as to unfit him for active duty, and would not mean that his case was dangerous, but although there were only forty-five cases sent to the hospital out of the thousand revaccinated Dr. Nash had no information about them. The more severe cases that required treatment would go into hospital, but not necessarily, if the man was simply unfit for duty (Qs. 3,609-17).

The experience of the Army Medical Department does not induce them to believe that even revaccination affords a complete immunity against small-pox, only relative immunity, and it is Dr. Nash's opinion that the deaths are fewer than they would be if there were not revaccination (Qs. 3,622-3). This relative immunity he thinks is shown by the number of attacks being less than they would be without vaccination (Q. 3,624). Being asked by Dr. Collins (Q. 3,625), "With what experience are you comparing it?" he answers, "With what I *have read** *of the times before vaccina-*

---

* The extraordinary exaggerations of the prevalence of small-pox in the past has been repeatedly and thoroughly exposed. Unfortunately, the advocates of vaccination rarely read anything opposed to their views It is true, however, that small-pox did become very prevalent through the medical practice of inoculating it. Of this, among an indefinite number of illustrations, the following will suffice: In 1792 there were 8,346 cases of small-pox in Boston, Mass., then a filthy city of less than 20,000 inhabitants, but 8,114 of these cases were cases of inoculated small-pox. It is probable that many, perhaps nearly all, of the remaining 232 cases were taken by

*tion. I have no figures here to go upon; it is my opinion.*" He has no figures to go upon. He has no figures to show the shortest time after successful vaccination with which small-pox has occurred or proved fatal (Q. 3,626).

Dr. R. D. R. Sweeting appeared before the Commission on the 13th November, 1889. He is Medical Superintendent of the Western Hospital (formerly called the Fulham Hospital), and has held that office since 1880. He had prepared some tables showing the protective value of vaccination as observed in small-pox cases treated at the hospital.

His first table (Q. 3,689) related to 2,584 cases of small-pox admitted into the hospital during the years 1880-85, and distinguishes between "good vaccination," "imperfect vaccination," "doubtful vaccination," and "no vaccination." He finds the cases under each category to be respectively 39, 1,921, 266, and 358, and the fatality to be none in the cases of good vaccination, 175 of imperfect, 88 of doubtful, and 165 in the cases of no vaccination, or the fatality per cent. in each case to be respectively none, 19.19, 33.08, and 46.08. Also, divided into age periods, by far the larger proportion of children admitted under five years were unvaccinated, and in these children small-pox was very fatal (Q. 3,701). The table also shows the fatality in each age period to be none among those classed under good vaccination, and to be less in each period in proportion to the vaccination.

It also shows that the proportion of imperfectly or doubtfully vaccinated cases to the unvaccinated rises greatly at the age period 20-30 as well as their fatality from 15 to 30 years, which Dr. Sweeting ascribes to "the waning protection of imperfect vaccination."

He also (Q. 3,717) introduced a table intended to show the effect of the number of *marks* upon the fatality at the

---

reason of the pestilential air induced by the 8,114 inoculated cases. (Report No. 153, House of Rep., Mass., March 13th, 1861, Section on small-pox, page 10.)

different age periods, and while he places the average fatality of the unvaccinated at 46.08 per cent., he finds that the vaccinated with one mark yield an average fatality of 10.37 per cent., with two marks 8.73 per cent., with three marks 7.45 per cent., with four marks 4.31 per cent., and with five or more marks 4.05 per cent.

Generally he states that the more marks the less fatality, but this does not apply at every age period (Qs. 3,718-20).

His next table (Q. 3,722) shows the fatality in 311 vaccinated cases, in which the scars were accurately measured, and shows that there were 60 cases with three deaths where the scar was more than half a square inch in area, and 251 cases with 40 deaths where it was less than half a square inch total area.

Up to the age of ten there were no deaths in these 211 cases, although none of those under ten had scars of more than half a square inch, and only 11 had scars of less than a half a square inch, and none of the 11 died, "so that," says Dr. Sweeting, "even vaccination falling short of the standard seems to be a considerable protection to children up to ten years of age; over ten years of age the numbers, both as regards attacks and deaths, seem to increase among those having less than half a square inch more rapidly than with those presenting more than half a square inch of scar, and taking them at all ages the fatality among those with more than half a square inch is 5 per cent., whereas with those with less than half a square inch it is nearly 16 per cent."

He presents another table (Q. 3,723) purporting to deal with the relation between the severity of small-pox and the observed condition of vaccination in "the 2,584 cases admitted into the hospital during the years 1880-85, and shows the proportion in each class of mild cases, namely, in the well vaccinated, nearly nine-tenths, in the imperfectly vaccinated four-fifths, in the doubtfully vaccinated about onefourth. On the other hand, in the 'not vaccinated' only 8 per cent. were mild cases." He has not been able to make a record of revaccinated cases; the evidence, as a rule, is very faulty (Q. 3,727). In very few cases were the marks obliterated by confluent small-pox when the patients entered the hospital, at which time the examination as to vaccination is made (Qs. 3,724-28).

(Q. 3,733) related to the hospital staff. Out of 362 persons 48 had had small-pox before they came to the hospital, seven took it while on the staff. Of these seven two were not revaccinated on entering the hospital. Of the ambulance staff, numbering 42, only one took small-pox, he was the coachsmith, who had not been revaccinated on entering the service. "He took small-pox thirteen days after he arrived on duty and died of the disease." Of the above 42 persons three had had small-pox before they came. "It is our practice to revaccinate all the staff immediately they come, and to repeat the operation if it is not successful; if it does not take after these two attempts then we let them take their chance" (Q. 3,734). There is not much failure in revaccination, "probably half of them take unmistakably."

(Q. 3,735.) Mr. Picton asked Dr. Sweeting how his alleged 46.08 fatality among the unvaccinated compared with the fatality before the introduction of vaccination, to which he replies: "There are no accurate data, I believe, as to prevaccinative fatality. The fatality at the London Small-pox Hospital at the end of the last century was, I believe, close on 30 per cent.; amongst hospital patients it would perhaps be higher than among the general population. *I am not aware of any trustworthy statistics of the actual fatality in prevaccination times.*"*

---

* This is an unkind slur upon such men as Dr. Jurin and other physicians, who, out of a very large number of cases of eighteenth century small-pox, concluded that the fatality of hospital cases was 18.8 per cent.; that of private cases was from 2 to 6 per cent.

He thinks the hospital patients come generally from the same district and are in the same class of life (Q. 3,732).*

Taking the London Small-pox Hospital fatality in pre-vaccination times at 30 per cent., he is unable to offer any explanation for the additional 16 per cent. now observed by him (Q. 3,740). He does not know whether small-pox has become more fatal in this century (Q. 3,742). He is not acquainted with the sanitary condition of the London Small-pox Hospital, nor is he aware that hospital gangrene was troublesome in its wards (Qs. 3,742-3).

His statistics as to "marks" differ from those of Marson (Qs. 3,746-7). "But I do not, however, deduct cases in which death might be referred to super-added disease."

He thinks that the unvaccinated and the vaccinated classes were strictly comparable statistically (Q. 3,750), but on being asked if he agrees with Dr. Gayton, who considers the unvaccinated to be drawn from a poorer class than the vaccinated, and that this would be likely to raise the unvaccinated mortality, he said: "I agree with him that the unvaccinated are drawn from a more neglected class, but I do not agree with him that that would necessarily raise their mortality from small-pox" (Q. 3,751).

(Q. 3,752.) Dr. Collins, quoting from the report of the Medical Superintendent to the Committee of the Fulham Hospital for 1879, on page 11, asks:

"Many of these unvaccinated cases were laborers driven from the country to the metropolis on account of the depression in agriculture and trade in search of occupation, and had unknowingly lodged themselves in infected haunts. Further, the number of unvaccinated children under five years during the year was the same (namely thirty-four) as during the year previous, and the high mortality in them must needs tell heavily on a smaller aggregate number of

* See below (Q. 3,751).

cases." "Do you think that would be a just criticism upon a comparison between vaccinated and unvaccinated mortality." *Ans.* "That is simply the opinion of Dr. Makuna, my predecessor."

(Q. 3,753.) "It does not agree with my own figures in the first table." (Q. 3,754.) "As regards the character of the patient I do not agree with what Dr. Makuna says, it does not agree with my experience at the hospital. I have no experience of unvaccinated cases lodging in infected haunts."

He considers (Q. 3,755) that there is no difference such as would operate upon small-pox mortality between the vaccinated and unvaccinated, except the facts of age and vaccination.

Apart from these two conditions (Q. 3,757), he considers the two classes "strictly comparable as regards attack,* as regards progress and course of disease, undoubtedly there might be a difference according to the state of life and previous history of the patient, antecedent circumstances unfavorable to health *might* have some effect upon the severity of the disease, but in my opinion would not have any effect upon the liability to attack."

He cannot tell whether his observed 329 severe cases out of a total unvaccinated of 358 would or would not be a large proportion as compared with prevaccination times (Qs. 3,678-79).

Vaccination gives decidedly less protection than a previous attack of small-pox (Q. 3,770).

(Q. 3,793.) He uses calf lymph for revaccinating the staff of the hospital; he found it more efficacious (Q. 3,795). He uses Dr. Warlomont's calf lymph, which comes from Brussels; he gets it from the association for the supply of lymph.

---

* What can be thought of a physician who considers the difference between good and malnutrition as of no account as regards liability to an attack of small-pox! Yet in the next sentence he entirely gives away his case with regard to fatality. Yet, even here, notice the "*might*" when will or *must* would be the proper word.

Dr. Sweeting does not know how he (Dr. Warlomont) obtained it originally (Q. 3,797).

He would rather not give his impressions upon the obscure subject of the relationship subsisting between cow-pox and small-pox (Q. 3,798). He has no statistics of the mortality in cases treated in the hospital ships and in the small-pox camp. He has not compared his experience with that experience (Qs. 3,802-3).

He considers his tables show an abiding protection against death by small-pox up to twenty or more years of age (Q. 3,817). There is a typical character about the vaccination mark (Q. 3,821) which there is not the least difficulty in recognizing (Q. 3,818).

Dr. Sweeting would not regard a person having only one mark as fully protected as a person with five marks. He might perhaps be as fully protected against attack, but not against death by small-pox (Q. 3,832).

It is not common for people to have five or more marks; there would not be a large proportion of them even in London. "We have had 148 people admitted with five or more marks" (Qs. 3,825-27).

(Q. 3,828.) "Do you think the column in your second table, 'Five or more marks,' indicates anything more than that there are but few people with five or more marks? Do you not think they had their fair proportion of small-pox—the 148 cases?" "It has not struck me that way."

